# Supplementary material for: Overexpression of Key Sterol Pathway Enzymes in Two Model Marine Diatoms Alters Sterol Profiles in Phaeodactylum tricornutum
Source: Pharmaceuticals (Basel). 2020 Dec 21;13(12):481. doi: 10.3390/ph13120481 (PMC7766473; doi:10.3390/ph13120481)
Supplement: Supplementary file 1 [file pharmaceuticals-13-00481-s001.pdf]

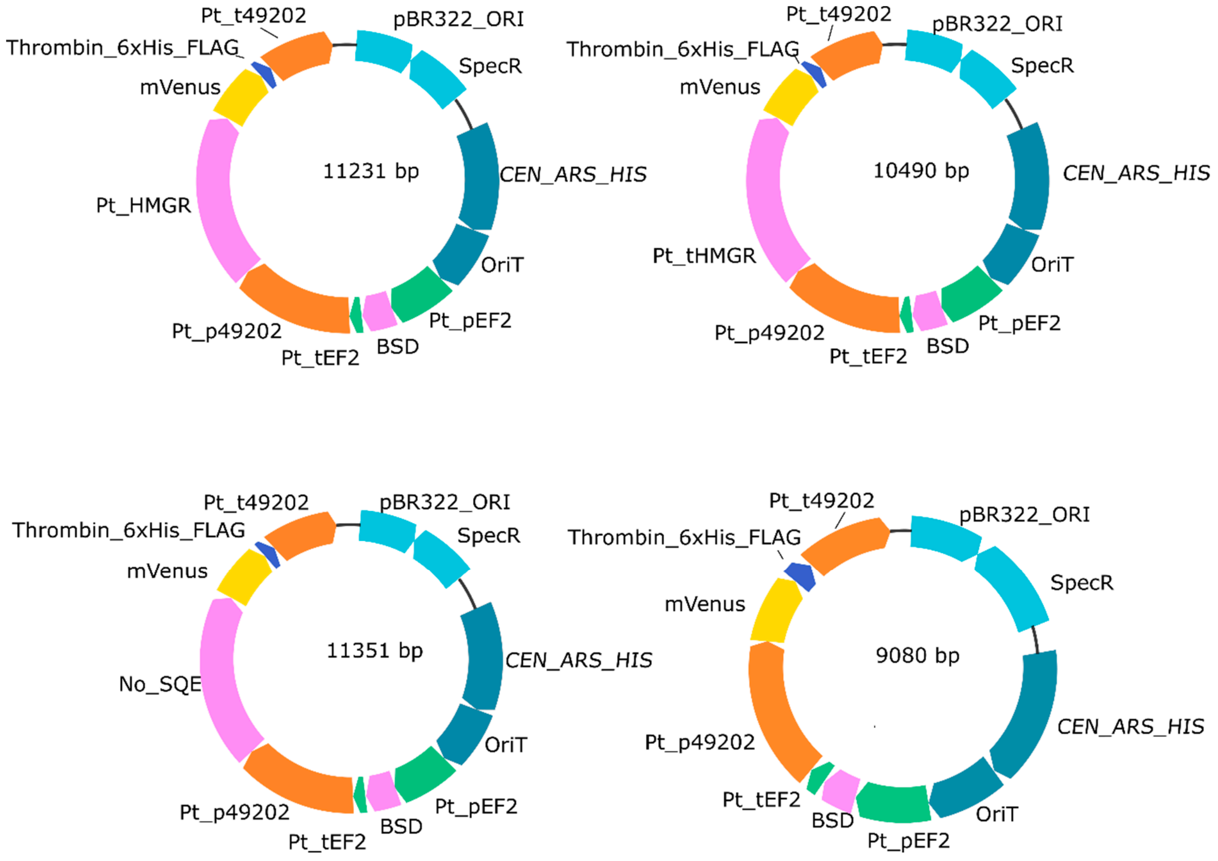

Figure S1. Maps of plasmids used for *P. tricornutum* transformation. CDS are shown in pink, in green promoters and terminators and fluorescence mVenus gene in yellow.

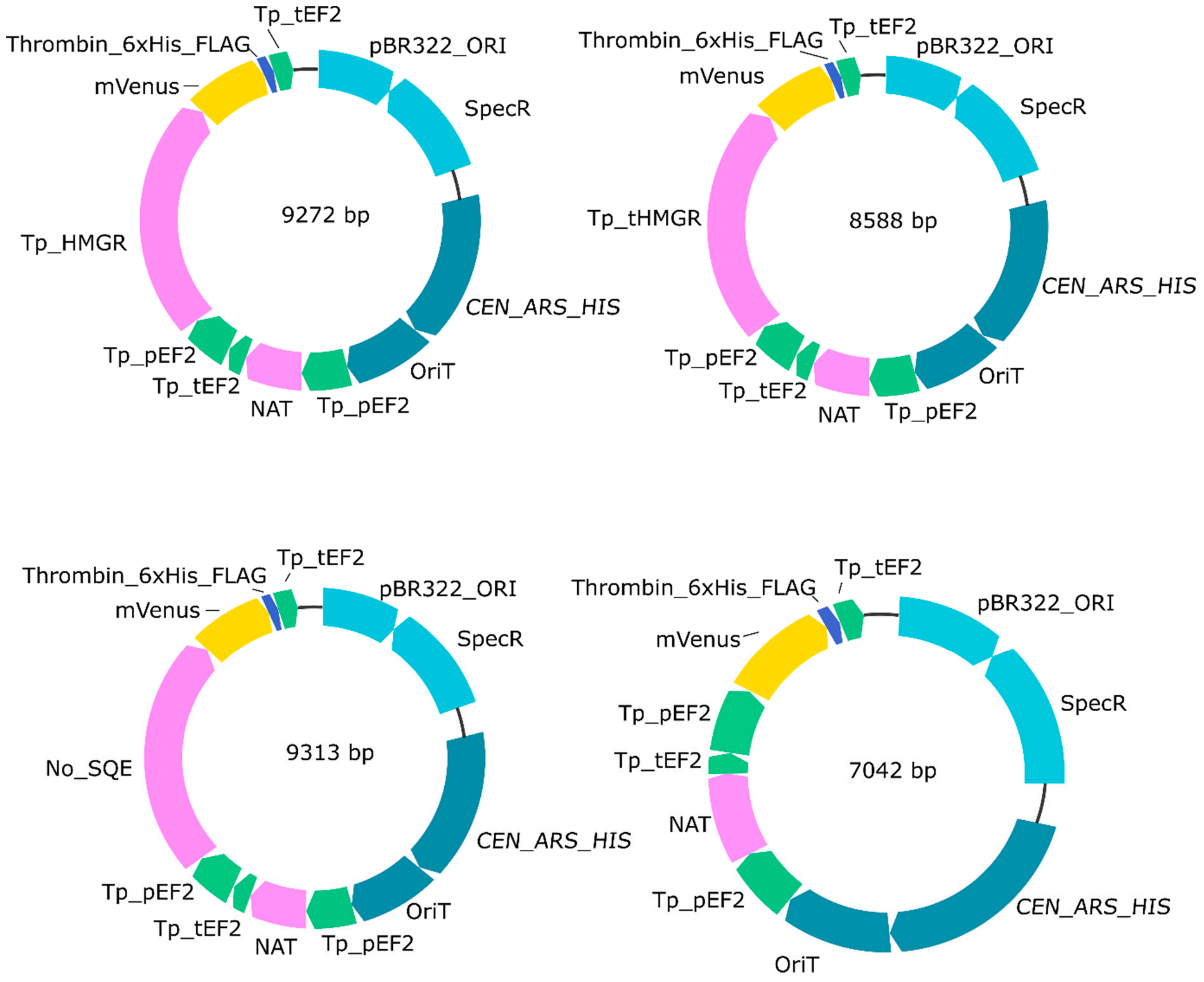

Figure S2. Maps of plasmids used for *T. pseudonana* transformation. CDS are shown in pink, in green promoters and terminators and fluorescence mVenus gene in yellow.

```
                                                                     *
Thalassiosira_pseudonana   311  VLGANCEIVVGYIPIPVGIVGPVTLNGESVYIPMATTEG-LVASTNRGCKAITQGSGARSTILRDGITRA
Phaeodactylum_tricornutum  286  VHGANCEIVVGYVPLPVGLVGPLTVNGETVYVPMATTEGCLVASTNRGAKAITAGGGATAVLLRDGITRA
Saccharomyces_cerevisiae   677  VFGACCENVIGYMPLPVGVIGPLVIDGTSYHIPMATTEGCLVASAMRGCKAINAGGGATTVLTKDGMTRG
Arabidopsis_thaliana       228  ILGQCCEMPVGYIQIPVGIAGPLLLDGYEYSVPMATTEGCLVASTNRGCKAMFISGGATSTVLKDGMTRA
Homo_sapiens               522  VMGACCENVIGYMPIPVGVAGPLCLDEKEFQVPMATTEGCLVASTNRGCRAIGLGGGASSRVLADGMTRG

Thalassiosira_pseudonana   410  FESTTSFGKLIEASPTVAGRNVYIRLRCFSGDAMGMNMISKGSLAVIECLREQF--PQLSLVALSGNMCT
Phaeodactylum_tricornutum  386  FESTTSFGKLLKCAPTVAGRNVYLRLTCFSGDAMGMNMVSKGSLAVIETLQQEF--PELVLVALSGNMCT
Saccharomyces_cerevisiae   777  FNSTSRFARLQHIQTCLAGDLLFMRFRTTTGDAMGMNMISKGVEYSLKQMVEEYGWEDMEVVSVSGNYCT
Arabidopsis_thaliana       328  FNRSSRFARLQSVKCTIAGKNAYVRFCCSTGDAMGMNMVSKGVQNVLEYLTDDF--PDMDVIGISGNFCS
Homo_sapiens               622  FDSTSRFARLQKLHTSIAGRNLYIRFQSRSGDAMGMNMISKGTEKALSKLHEYF--PEMQILAVSGNYCT
                                               *
Thalassiosira_pseudonana   508  TLKTSVPAIVEANVNKNLIGSAMAGTVGGFNAHAANNVTAVFLATGQDPAQNVESSNCITLMEVSP--EG
Phaeodactylum_tricornutum  484  TLKTTVHSMVQTNLHKNLIGSAMAGALGGFNAHASNIVTAVFLATGQDPAQNVESSNCITLLEETE--EG
Saccharomyces_cerevisiae   877  VLKSDVSALVELNIAKNLVGSAMAGSVGGFNAHAANLVTAVFLALGQDPAQNVESSNCITLMKEVD---G
Arabidopsis_thaliana       426  VLKTSVAALVELNMLKNLAGSAVAGSLGGFNAHASNIVSAVFIATGQDPAQNVESSQCITMMEAIN-DGK
Homo_sapiens               720  VLKTTTEAMIEVNINKNLVGSAMAGSIGGYNAHAANIVTAIYIACGQDAAQNVGSSNCITLMEASGPTNE
                                              *
Thalassiosira_pseudonana   606  AIGVKG-GGENPGDNARQLAHVVACATMAGELSLMAALASNSLVAAHMQHNRKPASK-------------
Phaeodactylum_tricornutum  582  AMGVRG-GGATPGAHAQKLAQIVASATLAGELSLLAALAANTLVQAHMQHNRKPAAK-------------
Saccharomyces_cerevisiae   974  LLGVRGPHATAPGTNARQLARIVACAVLAGELSLCAALAAGHLVQSHMTHNRKPAEPTKPNNLDATDINR
Arabidopsis_thaliana       525  LLGVKGASTESPGMNARRLATIVAGAVLAGELSLMSAIAAGQLVRSHMKYNRSSRDISGATTTTTTTT--
Homo_sapiens               820  MLGVQGACKDNPGENARQLARIVCGTVMAGELSLMAALAAGHLVKSHMIHNRSKINLQDLQGACTKKTA-
```

Figure S3. Sequence alignment of HMGR protein (catalytic domain) from model organisms.

Asterisks (*) indicate conserved catalytic residues.

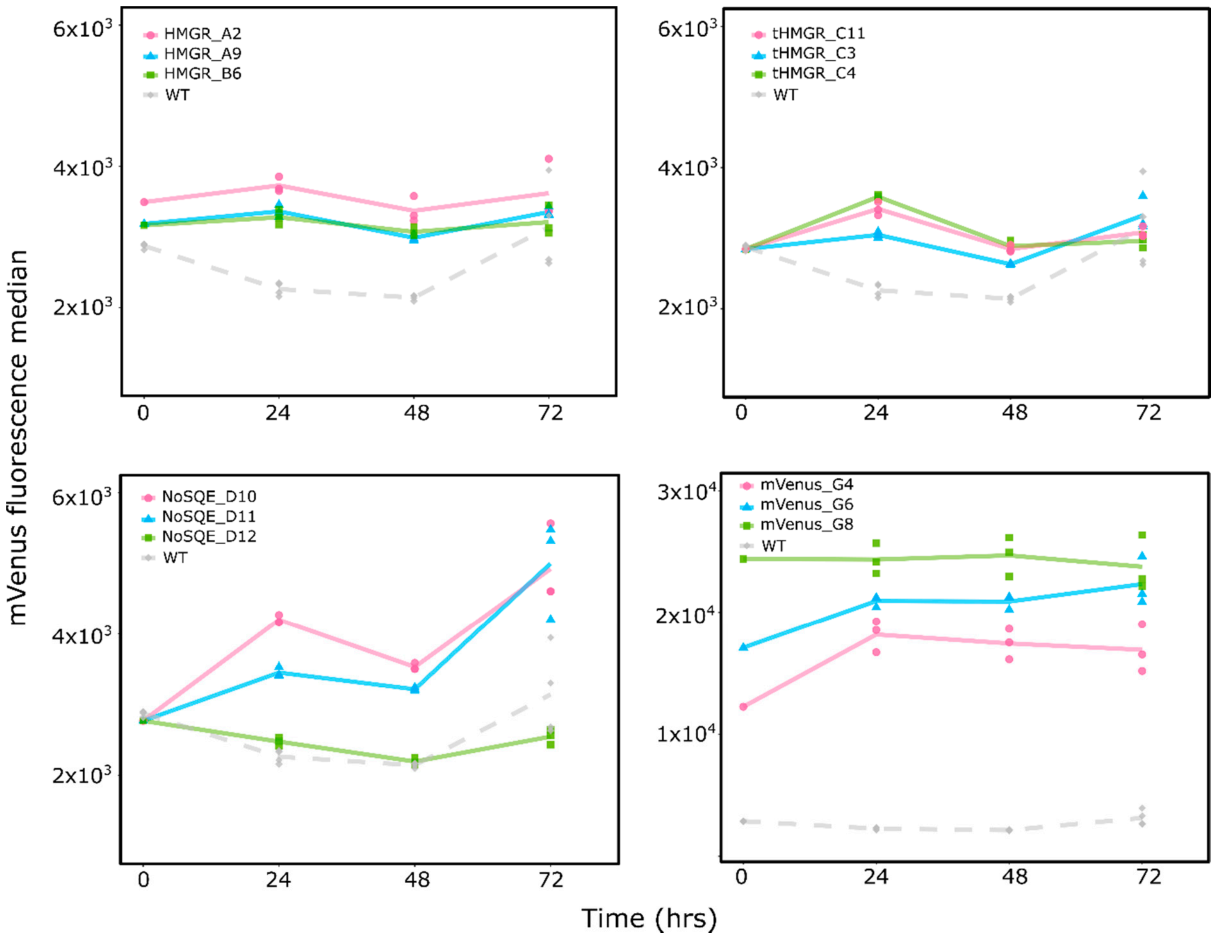

Figure S4. mVenus fluorescence during full scale experiment in *P. tricornutum* transformants (n = 3).

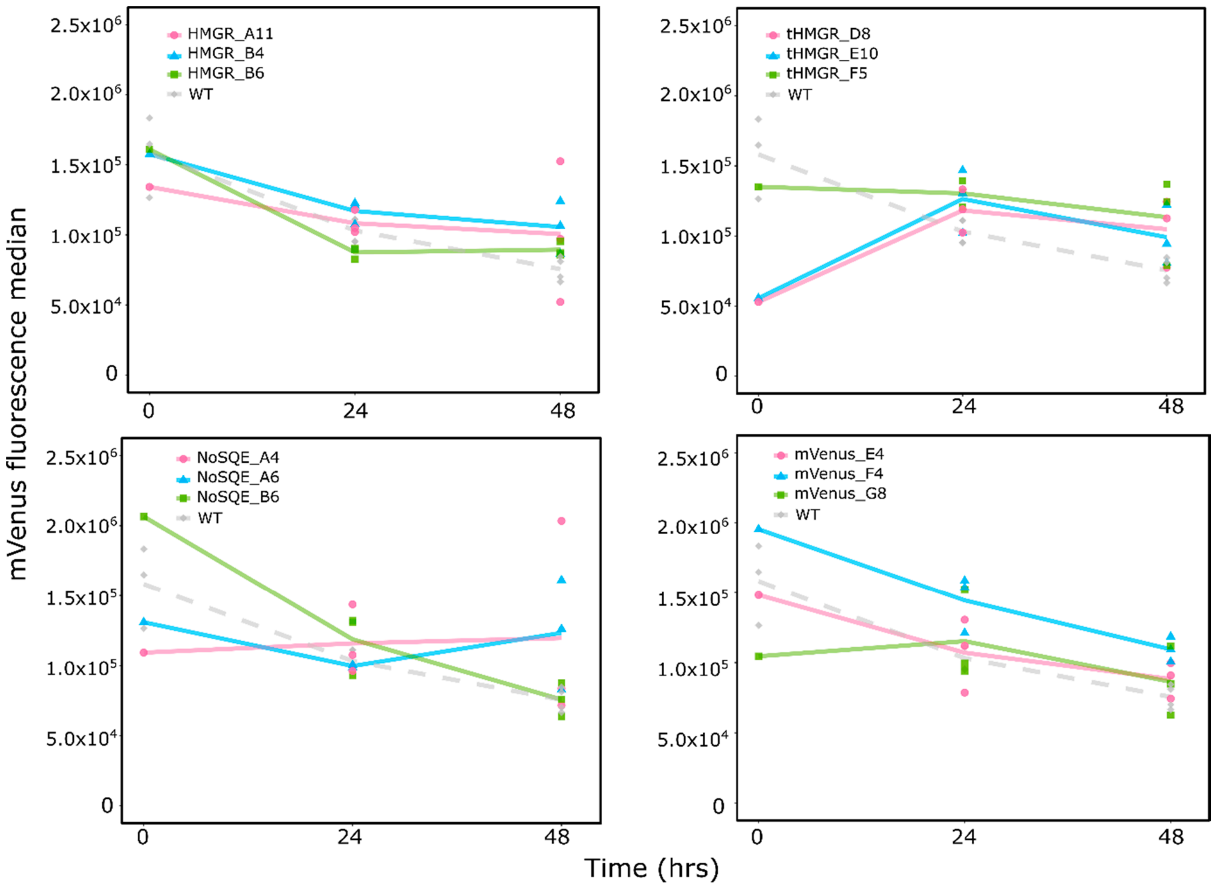

Figure S5. mVenus fluorescence during full scale experiment in *T. pseudonana* transformants (n = 3).

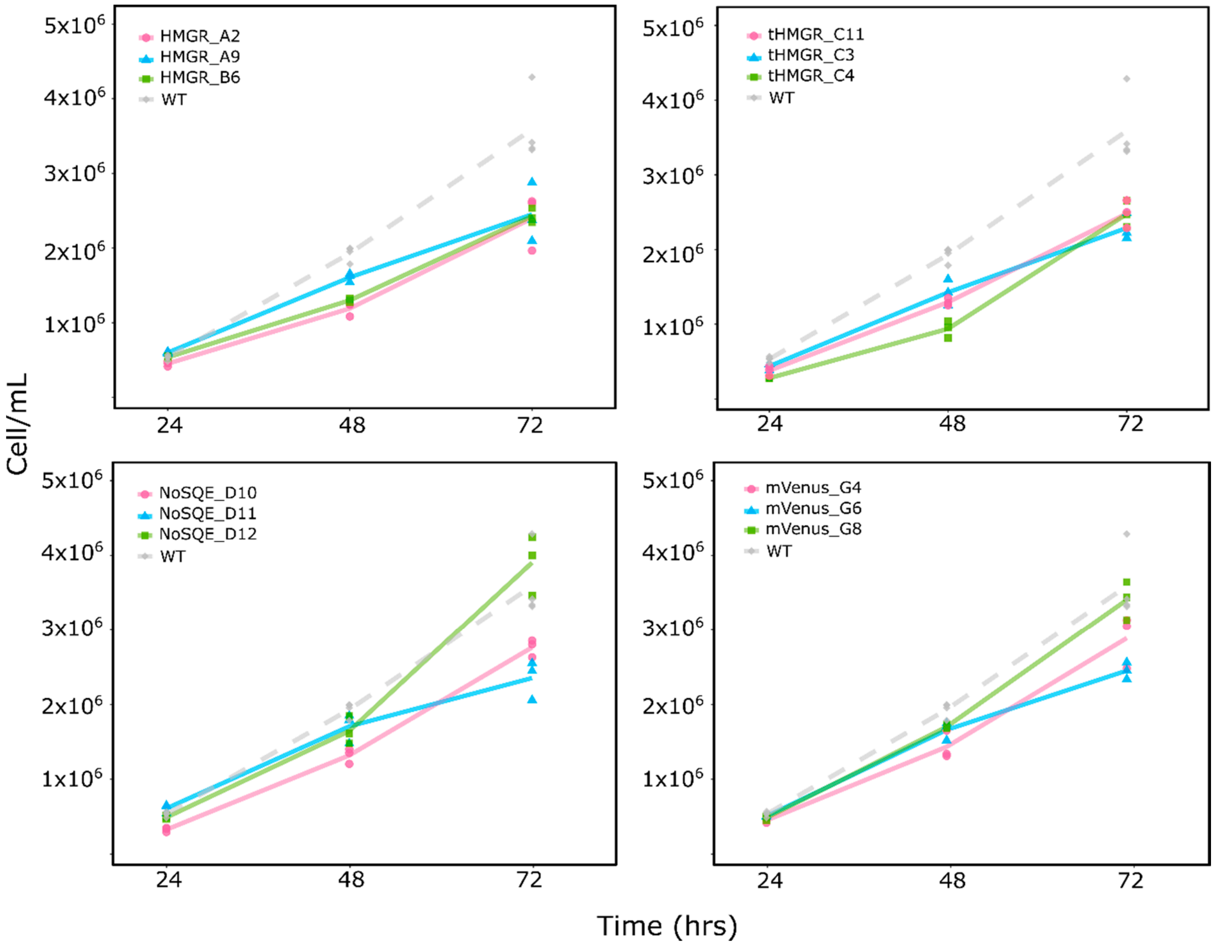

Figure S6. Growth curves during full scale experiment for *P. tricornutum* transformants (n = 3).

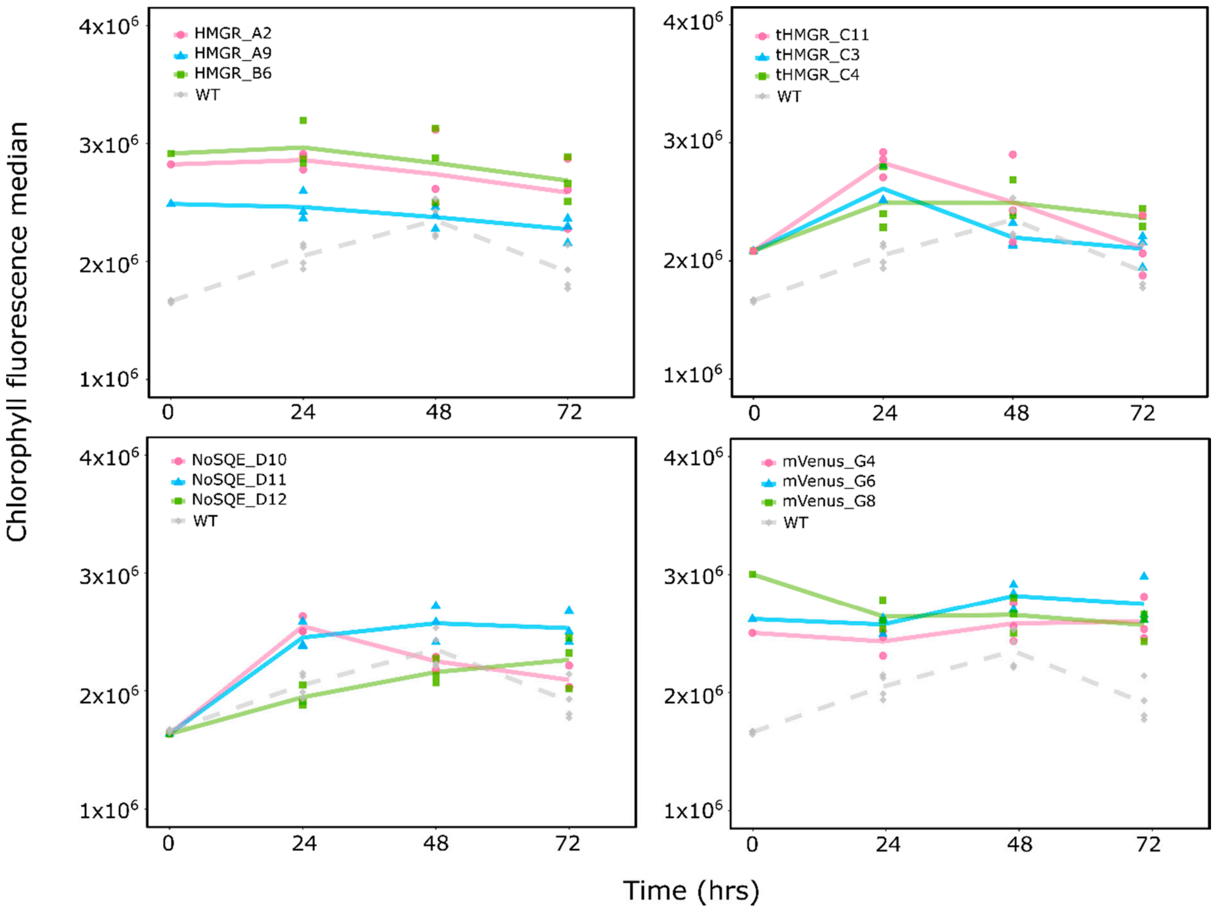

Figure S7. Chlorophyll fluorescence during full scale experiment in *P. tricornutum* transformants (n = 3).

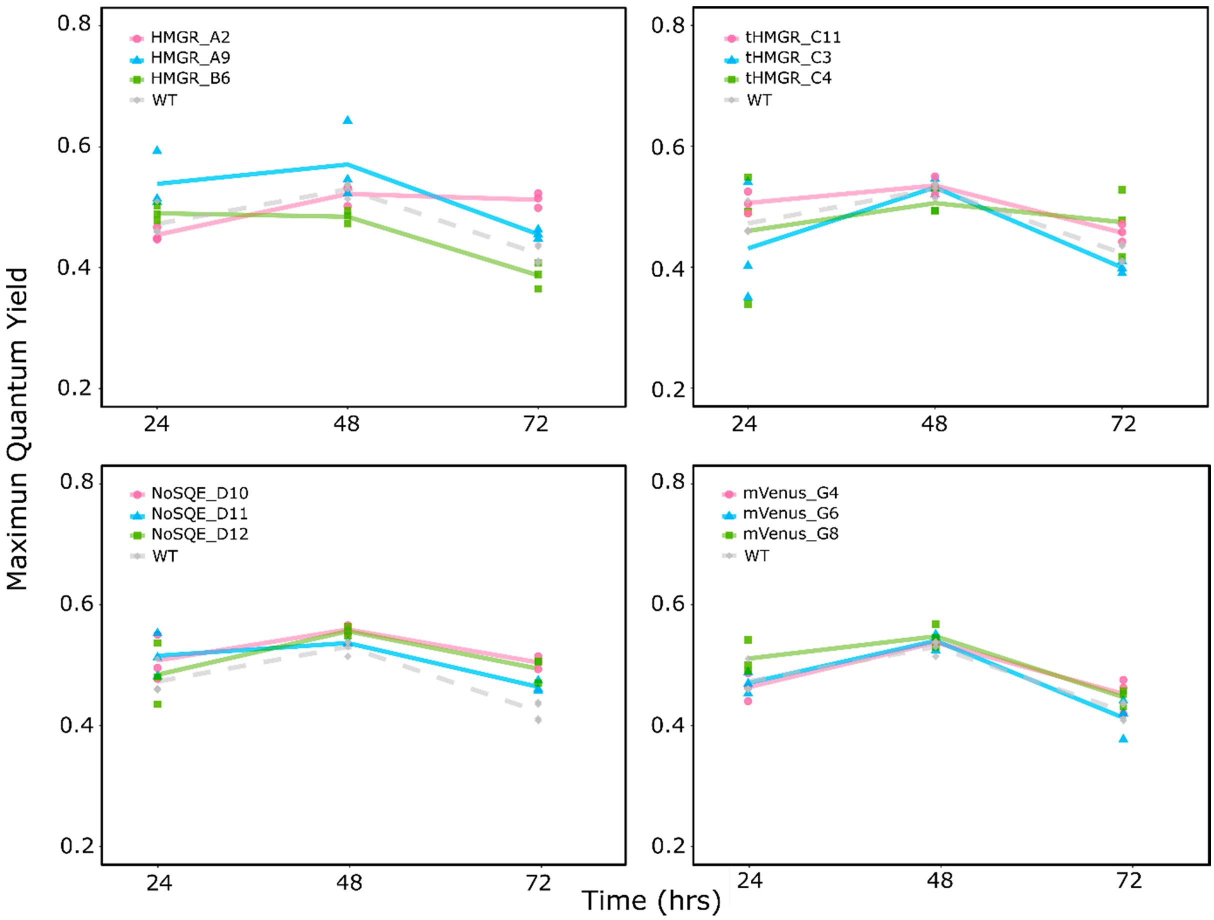

Figure S8. Maximum quantum yield for *P. tricornutum* transformants during full scale experiment (n = 3).

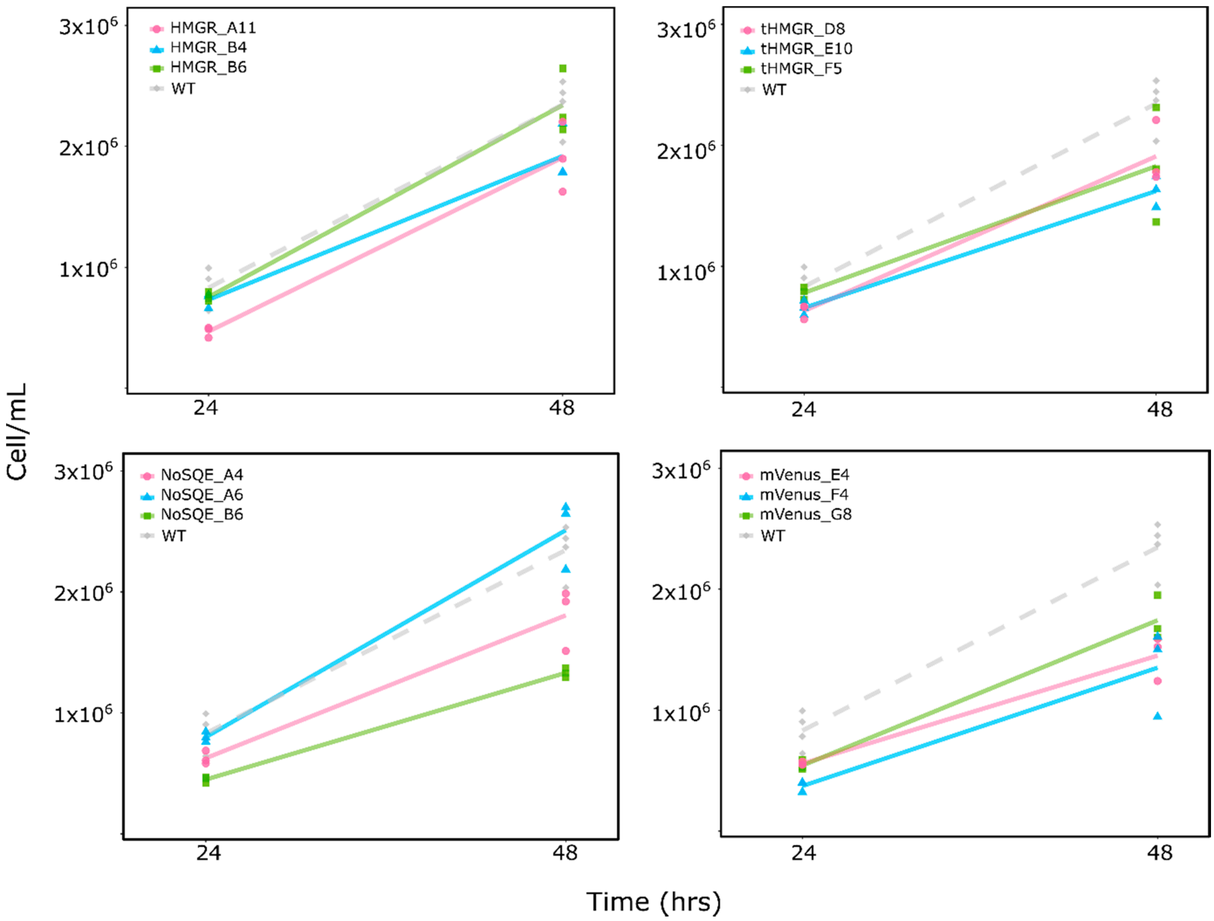

Figure S9. Growth curves during full scale experiment for *T. pseudonana* transformants (n = 3).

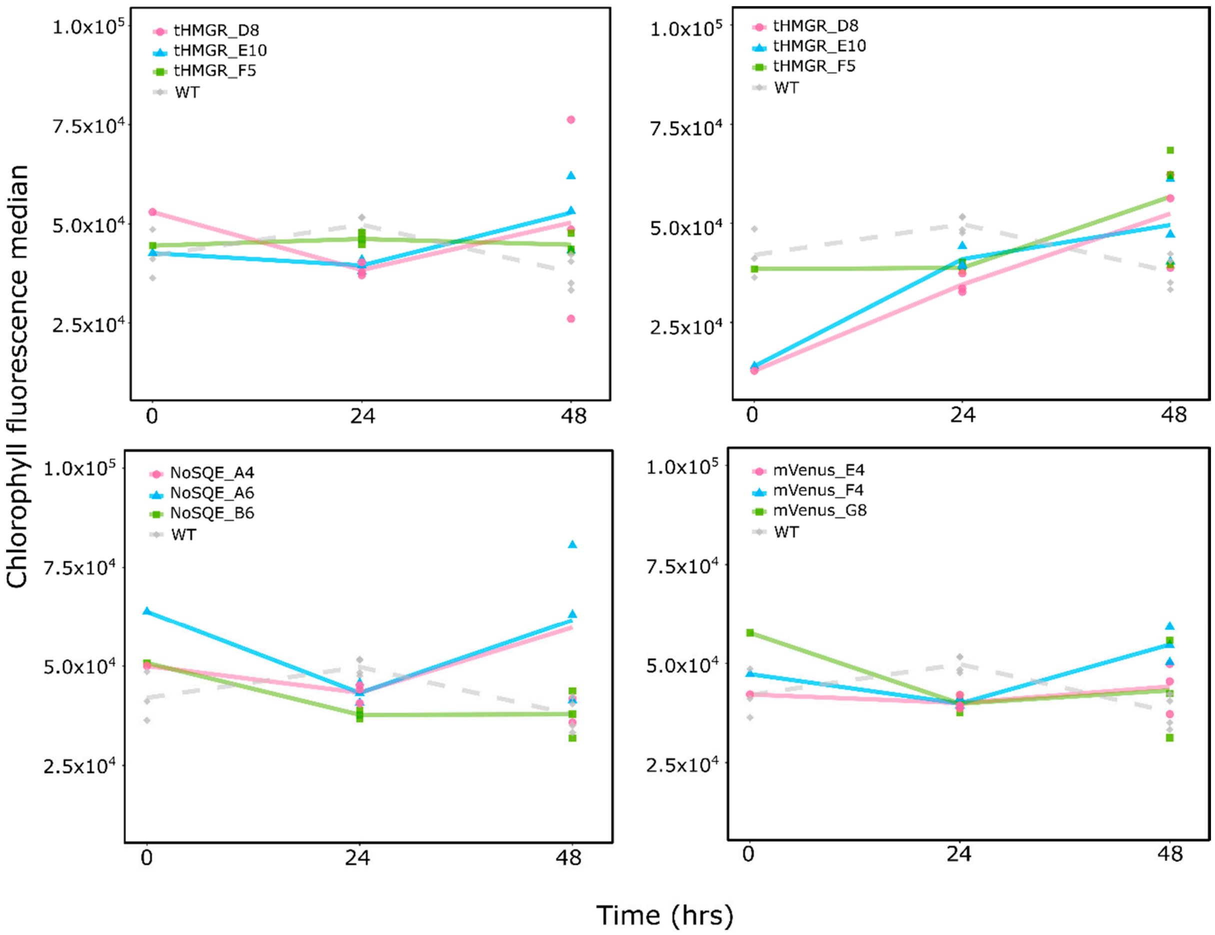

Figure S10. Chlorophyll fluorescence during full scale experiment in *T. pseudonana* transformants (n = 3).

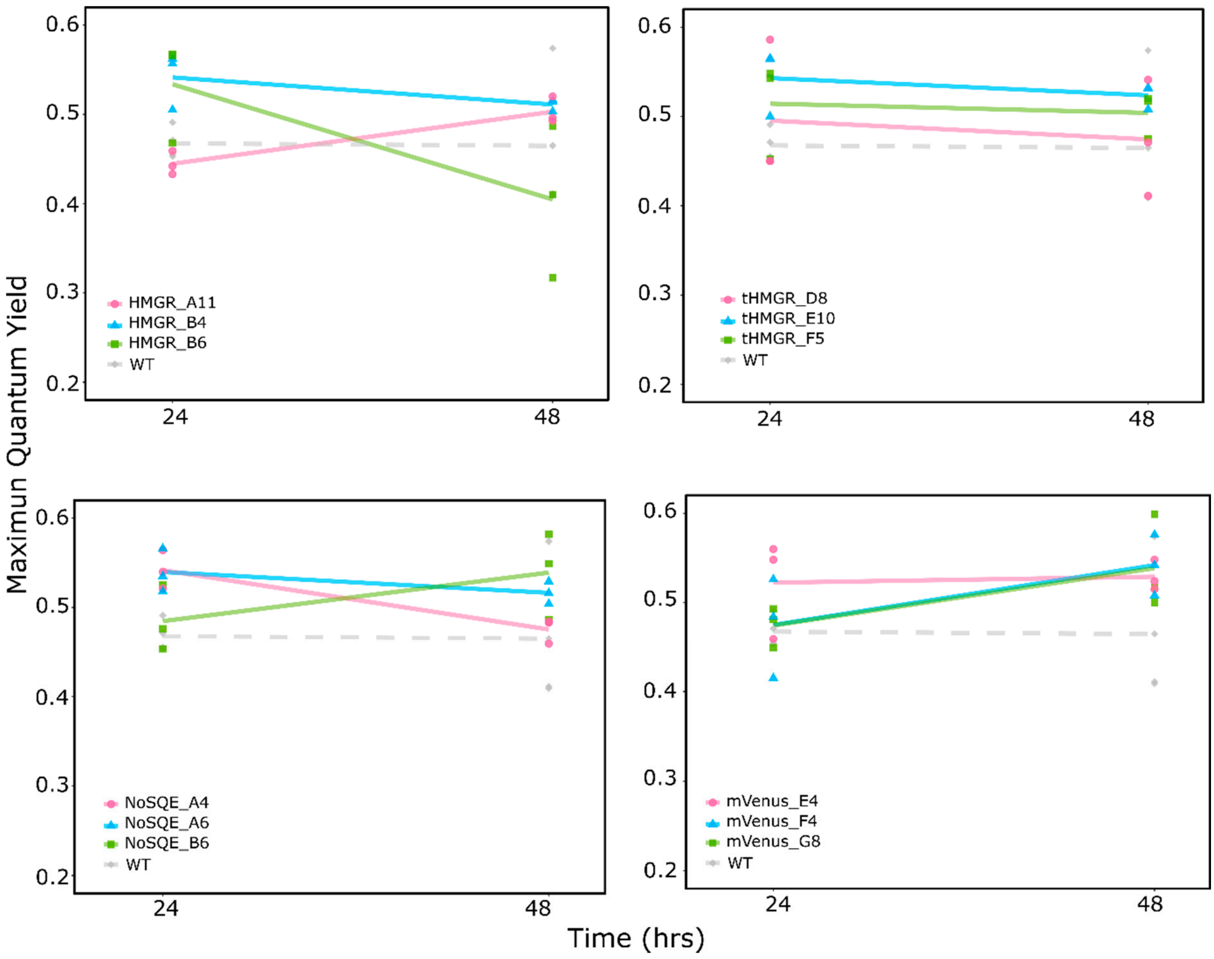

Figure S11. Maximum quantum yield for *T. pseudonana* transformants during full scale experiment (n = 3).

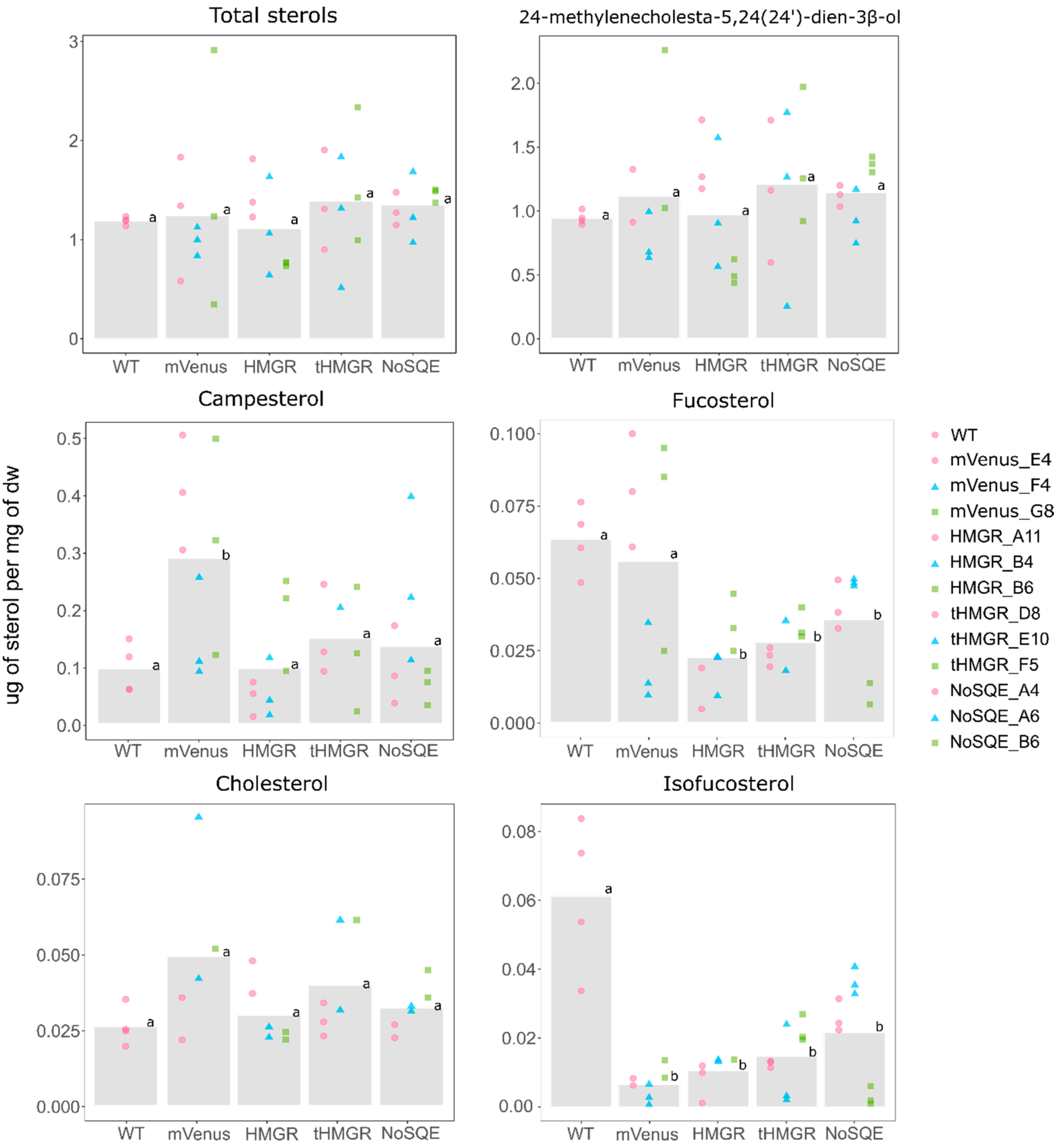

Figure S12. Sterol levels in *T. pseudonana* transformants. Identical letters denote no statistically significant differences among groups using the Pairwise Wilcoxon Rank Sum tests ($p < 0.05$, n = 9).

Table S1 L0 parts for construction of episomes using uLoop assembly method [62]. Primers sequences used for domestication are presented for L0 each part

L0 part	Gene ID	Description	Template	Primers	Plasmid number
AC_pTpEF2	269148	Elongation factor promoter	*P. tricornutum* genomic DNA	F: AAGCTCTTCATCCggagAGTGTGCAATGCAGTCAATTCAATAGATATG<br>R: TTGCTCTTCTTCGcattCTTGACGTTCTTTTCTCTTTAATTAATCGCG	
AC_pPtEF2	Phatr3_J35766	Elongation factor promoter	*P. tricornutum* genomic DNA	F: AAGCTCTTCATCCggagTCCATTTTGACATGTTTCCTAGCTAGAAG<br>R: TTGCTCTTCTTCGcattTGTGTGGAGAGAACGAGCAGCAGCG	
AC_pPt49202	Phatr3_J49202	Predicted protein JCVI	NA	NA	
AC_CENARSHIS	NA	CEN6-ARSH4-HIS3 episome maintenance sequence from PtPBR11 (Genebank KX523203) (Diner *et al.*, 2016)	NA	NA	
CF_OriT	NA	Origin of transfer, originally called basis of mobilization (Diner *et al.*, 2016)	PtPBR11 (Genebank KX523203)	F: CAGAAGCTCTTCATCCaatgGATCGTCTTGCCTTGCTCGTCGG<br>R: TTGCTCTTCTTCGagcgATCTTCCGCTGCATAACCCTGCTTCGG	139948
CD_NoSQE	521007	0.0	NA	NA	
CD_TpHMGR	33680	3-hydroxy-3-methylglutaryl-coenzyme A reductase (EC: 1.1.1.34)	*T. pseudonana* genomic DNA	F: AAGCTCTTCATCCaatgGCGGCAGCAGCAGCACCAACCATAG<br>R: TTGCTCTTCTTCGacctgaCTTTGAAGCAGGCTTGCGATTATGTTG	
CD_TptHMGR	33680	Truncated 3-hydroxy-3-methylglutaryl-coenzyme A reductase (EC: 1.1.1.34)	*T. pseudonana* genomic DNA	F: AAGCTCTTCATCCaatgAGCCCCAACGACCCACCCGTCAAAG<br>R: TTGCTCTTCTTCGacctgaCTTTGAAGCAGGCTTGCGATTATGTTG	
CD_PtHMGR	Phatr3_J16870	3-hydroxy-3-methylglutaryl-coenzyme A reductase	*P. tricornutum* genomic DNA	F: AAGCTCTTCATCCaatgACGGTGACTATCAGCAGTAGTATTAG<br>R: TTGCTCTTCTTCGacctgaCTTGGCGGCGGGTTTGCGGTTG	
CD_PttHMGR	Phatr3_J16870	Truncated 3-hydroxy-3-methylglutaryl-coenzyme A reductase	*P. tricornutum* genomic DNA	F: AAGCTCTTCATCCaatgGACTCTATTTCCACCAAGACCAGCGCG<br>R: TTGCTCTTCTTCGacctgaCTTGGCGGCGGGTTTGCGGTTG	

CD_bsd	NA	blasticidin-S deaminase	pST1374-N-NLS-flag-linker-Cas9-D10A (Addgene #51130)	F: AAGCTCTTCATCCaatgGCCAAGCCTTTGTCTCAAGAAGAATCCAC<br>R:TTGCTCTTCTTCGacctgaGCCCTCCCACACATAACCAGAGGG	
CD_nat	NA	nourseothricin acetyltransferase	pAGM4723:TpCC_Urease (Addgene #85982)	F: AAGCTCTTCATCCaatgACCACTCTTGACGACACGGCTTACCGG<br>R:TTGCTCTTCTTCGacctgaGGGGCAGGGCATGCTCATGTAGAGCG	
CD_mVenus		Yellow fluorescent protein	NA	NA	
DE_Thr6XHisFLAG	NA	General C-terminal protein tag. Thormbine, 6xHIS and FLAG (DYKDDDDK) tag	DE_Venus-ThrHISFLAG L0 part	F: AAGCTCTTCATCCaggtGCGCTGGTCCCTCGCGGTAG<br>R: TTGCTCTTCTTCGaagcTCACTTATCGTCATCATCCTTGTAGTCG	
DE_Venus-ThrHISFLAG	NA	Yellow fluorescent protein with a Thormbine, 6xHIS and FLAG (DYKDDDDK) tag JCVI	NA	NA	
DE-3xStop	NA	Three stop codon	NA	NA	
EF_tPtEF2	Phatr3_J35766	Elongation factor terminator	*P. tricornutum* genomic DNA	F: AAGCTCTTCATCCGCTTACAGAAAAACAGACTCATAGGGTAC<br>R:TTGCTCTTCTTCGAGCGGAGACATTACTCCACACGATGG	
EF_tTpEF2	269148	Elongation factor terminator	*T. pseudonana* genomic DNA	F: AAGCTCTTCATCCgcttATATCTTCTTGCAACAATGGTAGCG<br>R: TTGCTCTTCTTCGagcgAGCAGGGTTGGTTAGAGATAACTAATG	
EF_tPt49202	Phatr3_J49202	Predicted protein	NA	NA	

Table S2. Source and number of transmembrane domains of *HMGR* sequences used for phylogenetic and domain analysis

Organism	MMETSP ID	Present	Source	Strain	Gene ID	TM*
Attheya septentrionalis	MMETSP1449	Y	Transcriptomics	CCMP2084	Transcript_1390	3
Chaetoceros affinis	MMETSP0091	Y	Transcriptomics	CCMP159	Transcript_17836	0
Chaetoceros brevis	MMETSP1435	N	Transcriptomics	CCMP164	NA	NA
Chaetoceros curvisetus	MMETSP0716	N	Transcriptomics	Unknown	NA	NA
Chaetoceros debilis	MMETSP0149	N	Transcriptomics	MM31A-1	NA	NA
Chaetoceros dichaeta	MMETSP1447	Y	Transcriptomics	CCMP1751	Transcript_4115	0
Chaetoceros muelleri	NA	N	Transcriptomics	CCMP1316	NA	NA
Chaetoceros neogracile	MMETSP0752	Y	Transcriptomics	CCMP1317	Transcript_689	3
Chaetoceros sp.	MMETSP1429	Y	Transcriptomics	UNC1202	Transcript_18209	0
Coscinodiscus wailesii	MMETSP1066	Y	Transcriptomics	CCMP2513	Transcript_2372	3
Cylindrotheca closterium	MMETSP0017	Y	Transcriptomics	KMMCC:B-181	Transcript_33356	3
Ditylum brightwelli	MMETSP1001	Y	Transcriptomics	GSO105	Transcript_14074	3
Extubocellulus spinifer	MMETSP0696	Y	Transcriptomics	CCMP396	Transcript_715	3
Fistulifera solaris	NA	Y	Genome	JPCC DA0580	GAX29119.1	3
Fragilariopsis kerguelensis	MMETSP0909	Y	Transcriptomics	L2-C3	CAMPEP_0196091342	2
Homo sapiens	NA	Y	Genome	NA	3156	5
Leptocylindrus danicus	MMETSP0321	Y	Transcriptomics	B650	Transcript_1399	2
Nitzschia punctate	MMETSP0747	Y	Transcriptomics	CCMP561	Transcript_27671	3
Nitzschia sp	MMETSP0014	Y	Transcriptomics	RCC80	Transcript_11673	3
Odontella Sinensis	MMETSP0160	Y	Transcriptomics	Grunow 1884	Transcript_15934	3
Oryza sativa	NA	Y	Genome	NA	LOC_Os02g48330	0
Phaeodactylum tricornutum	NA	Y	Genome	CCMP632	Phatr3_J16870	3
Pseudonitzschia arenysensis	MMETSP0329	Y	Transcriptomics	B593	CAMPEP_0116128146	3
Pseudonitzschia delicatissima	MMETSP0327	Y	Transcriptomics	B596	CAMPEP_0116102328	3
Pseudonitzschia multiseries	NA	Y	Genome	CLN-47	288249	3
Pseudonitzschia pungens	MMETSP1061	Y	Transcriptomics	cf. pungens	Transcript_13267	3
Rhizosolenia setigera	MMETSP0789	Y	Transcriptomics	CCMP1694	CAMPEP_0178949462	3
Saccharomyces cerevisiae	NA	Y	Genome	ATCC 204508	854900	7
Skeletonema marinoi	MMETSP1428	Y	Transcriptomics	UNC1201	Transcript_20411	3
Skeletonema menzelii	MMETSP0603	Y	Transcriptomics	CCMP793	Transcript_7316	3
Solanum lycopersicum	NA	Y	Genome	NA	Solyc02g082260.3	2
Thalassiosira miniscula	MMETSP0737	Y	Transcriptomics	CCMP1093	Transcript_23358	2
Thalassiosira oceanica	NA	Y	Genome	CCMP1005	91521	2
Thalassiosira pseudonana	NA	Y	Genome	CCMP1335	269148	3

Thalassiosira puntigera	MMETSP1067	Y	Transcriptomics	Tpunct2005C2	Transcript_37597	3
Thalassiosira rotula	MMETSP0403	Y	Transcriptomics	CCMP3096	Transcript_15672	2
Thalassiosira weissflogii	MMETSP1414	Y	Transcriptomics	CCMP1010	Transcript_6235	3
Thalassiothrix antarctica	MMETSP0152	Y	Transcriptomics	L6-D1	Transcript_7954	3

*TM indicates number of transmembrane domains predicted by TMHMM Server v. 2.0 (http://www.cbs.dtu.dk/services/TMHMM-2.0/). One of the three transmembrane domains in Arabidopsis is located in C-terminus domain.

HMGR sequences for phylogenetic reconstruction

>Thalassiosira_pseudonana
MAAAAAPTIGQRLDTLLAALSNIGDMNHLLSNYQPSTSQIYSLVIVLSVAFSFRLLNSGDDLGSKLSSSTVEQ
AKRGEKGTSSKYAKANNKNNKQSYDFPQPKWHILKLTNYVVTTLFLLSIITFLSNASVYLNDNTALMTFLGV
WSGLLCYFFGFFGISFVELDDLVVTDADGQQRQQGVVTQPQKQASCKSRKVESPNDPPVKVISLHPPASS
TPVCSDPIKSKSPTNTGIPTNVKDLSNEEIATLVLQDKIKDHQLEKLLDPHRAVAVRRLKFDALLSSLGKTTGE
DDKKGGVLSELPHEHDLDYKRVLGANCEIVVGYIPIPVGIVGPVTLNGESVYIPMATTEGLVASTNRGCKAI
TQGSGARSTILRDGITRAPCVRLPSAHEAAQVHLWIEEADNFAKLKEAFESTTSFGKLIEASPTVAGRNVYIR
LRCFSGDAMGMNMISKGSLAVIECLREQFPQLSLVALSGNMCTDKKAAAMNWIEGRGKSVVIEATIPKD
VVRSTLKTSVPAIVEANVNKNLIGSAMAGTVGGFNAHAANNVTAVFLATGQDPAQNVESSNCITLMEVS
PEGDLWISCTMPSIEVGTVGGGTGLSAQSACLRAIGVKGGGENPGDNARQLAHVVACATMAGELSLMA
ALASNSLVAAHMQHNRKPASK
>Phaeodactylum_tricornutum
MTVTISSSISSSIGSSTTAPTLGMQVDALVQQMDNLSSTQLYGLIVGLTILVSFVLLGSSADIPVSLRDT
TKETSSSSSTPRKTTTVSSSSNRGPEPRWHIFTYVNYAIVACFVASVAEFGRNASAYLAADDNVVLYFLV
AWSVFLCYFFGFFGVSFVHDADAAVASPTPTKPTVRDSISTKTSASNTLSARHPPAPSAPVCSDPSSFTP
IHTSKTNITTLDNAAICQLVLTNQIKDHELEKRLDAHRAVQVRRLVVAHKLDTLEHINAHALDNLPSEPS
LDYTRVHGANCEIVVGYVPLPVGLVGPLTVNGETVYVPMATTEGCLVASTNRGAKAITAGGGATAVLLRD
GITRAPCVRMPSAAQAAHLKLWCETPQHFSTLKRAFESTTSFGKLLKCAPTVAGRNVYLRLTCFSGDAMG
MNMVSKGSLAVIETLQQEFPELVLVALSGNMCTDKKAAATNWLEGRGKSIVVEATIPKDVVTNTLKTTVH
SMVQTNLHKNLIGSAMAGALGGFNAHASNIVTAVFLATGQDPAQNVESSNCITLLEETEEGDLWISCTM
PSIEVGTVGGGTSLPAQAACLQAMGVRGGGATPGAHAQKLAQIVASATLAGELSLLAALAANTLVQAHM
QHNRKPAAK*
>Fistulifera_solaris
MESLRLQLSQALSSFVSVSSNQLYGVIVGVTVFGCWGILSLAEQPELPEWQKTAVQRVPRQKEGPEPQW
HLFRWINMLVVVAFACSVAEFSLNATAYDSNVLCQFLMAWSLLLCYFFGFFGVMFVHDLEDSETVSTPVT
KSSKIHPPASSTPICSDAIQSKQNKLLIIEDIKDKKDAEIAQLVFTNQIKDHELEKQLDCHRAVTVRRLVVEQKL
ASLGHSGALEGLPYETDLDYSRVHGANCEIVVGYVPLPVGIIGPLTINGESFYIPMATTEGCLVASTNRGAKA
ITEGGGALAQIVRDGITRAPCIRMPTAMAAAQLKLWCETPENFVQMKAAFESTTSFGKLHECKATIAGKN
VYLRLVCFSGDAMGMNMVSKGTLAVIECLRKEFPELSLVALSGNMCTDKKAAATNWLEGRGKSVVVEAT
IPKDVVRKTLKTTVPAIVETNLNKNLVGSAMAGVIGGFNAHASNIVSAIFLATGQDPAQNVESSNCITLMEE
TESGDLWISCTMPSIEVGTVGGGTSLAAQAACLKALGCQGGGANPGDNAQRLAKVIASATMAGELSLLA
ALAANTCTYLLTDGFWRTNINSPIPSFFSVVAAHMQHNRKPTK
>Pseudonitzschia_multiseries
MAETMGTMPTLGMRLDNMIAHIDQIPSTQIYGLIVAATVGLCVVLLGTGNSNLEIHHQRQMRLQQQSQ
NDDLKKPKMAASARSGKQPKWHLFKWINFVAVGGFLYSVFTFCSNASRYLHHESQGVLVQFLVGWSIFL
MYFFGFFGVSLIHEDIPREEEESASIPSDPSSFKTSKASSSIPENLKELGNDEIAALVLDNKVKDHMLEKLLD
PFRAVTVRRIACNQKLAAVLGRNNNDTNVLDKLPSEPSLDYSRVFGANCEIVVGYVPLPVGLVGPLTIND
ESVYVPMATTEGCLVASSNRGAKAITQGGGARARIVRDGITRAPCLRMNSAMEAADLKIWCELPQNFAIL
KQAFESTTSFGKLLECNPTVAGKNVYLRLVCFSGDAMGMNMVSKGSLAVIETLQKQFPTCQLIALSGNMC
TDKKAAATNWLLGRGKSVVVECVIPKEVVRTTLKTTVAALVHTNLNKNLIGSAMAGAIGGFNAHASNIVT
AVFLATGQDPAQNVESSNCITLMEEQDDGDLWMCCTMPSIEVGTVGGGTSLPAQAACLEAIGCKGGGS
TPGANAKKLATVVAAATMAGELSLLAALAANTLVQAHMVHNRKPAAKK*
>Thalassiosira_oceanica

MMAGTATIGQRLDALVATAQANTDGSAALFALVIGISVSFSFYLLNSGSGSTASAEMTKCGAQLSTCPKD
NRTAIQKPRSPEDPPEPNWNALKLTNCVAATGFLLSVLKFASNASAYLNDSTSLLQFLSIWSVFLLYFFG
FFGIALVDLDGLVEEQPSPAPAAKSRQPISPSKKIAKTVDVEPSPPVKVVNVHPPAKSAPVCSDPSSLKR
HASSVPSNLKEMPNEEIAALVLQDKIKDHQLEKLLDPHRAVEVRRLKFDAKLKSLGNGGALNDLPHKHDL
DYKRVLGANCEIVVGYVPIPVGLAGPITLNGESVYIPMATTEGCLVASTNRGCKAITQGSGAFSTILKDG
ITRAPCCRLPSAQQAAEVAIWIETPENFAELKRAFESTTSFGKLIEARPTVAGRNVYIRLKCFAGDAMGM
NMISKGSLAVIECLRSIFPALSLLALSGNVCTDKKAAAMNWIEGRGKSVVIEATIPKDVVSKTLKTSVKA
IVDANVNKNLIGSAMASVVGGFNAHAANNVTAVFLATGQDPAQNVESSNCITIMEETSDGNLWVSCTM
PSIEVGTVGGGTGLPAQAACLRAIGVKGGGEKPGQNAKQLAHVVAAATLAGELSLMAALASNSLVAAHM
AHNRKPPTASKVEKAFDEVQHGFDDVWLSGQQQHGKKLLDRCSHPHKNWLLGLLIKLQARLRSRLTVAV
GLAPRPQDHRYFIGITRYAPLGPIIGISSALRVMRLWDQSRPKQIWQRFLTDPTQTPYTIHDQLLGLTLVLKS
AKNLQKSEMTPAAERRGSINRGYRITDSTCGFCSTEVCEWITDRLDHDNWDTCSGQRQGASWTNNTAI
QSLVVGGQEPARAPLYLHYVPRLYKAHARSKITGVVCFRLQIDPLSYASWR
>Extubocellulus_spinifer
MTTVPTLGERLDALVDRIDSIPSTQLYIAAVVTSVVFSCMLLNSGGSNGVVDPAKIPDNI
GRPPSVRRKRRDGSNPEDEPEPRWHILTILNYAAVTSFTSSVLYFATDASRFLADSSSLL
KFITGWSVFLCYFFGFFGISFVDADGLIAREEHEAAARQQQRMQKQHSLGISSPPSKSK
SKLHSAACSVPVSSDGASTKKRSSSSSCTPVDVKELADEKIASLVLTGQIKDHTLEKILG
CDRAVTVRRLAFDCKLSTLDRGGALSDLPSGPSLDYDRVFGANCEVVVGYIPIPVGMVGP
LTLNDETFFVPMSTTEGCLVASTNRGCKAISQGTGAISTVIRDGITRAPCVRMKSAKEAA
DLKIWCEQKDNFALLKKEFESTTSFGKLLSVSPIVAGKNVYIRLQCFSGDAMGMNMVSKG
SLAVIDLLKSTFPTLELVALSGNVCCDKKSGAINWIEGRGKSVVVEATIPLEVVRSTLKT
NVKTIVHVNIQKNLIGSAMASTVGGFNAHASNIVTAIFLATGQDPAQNVESSNCITLMEE
TDEGDLWISCTMPSIEVGTVGGGTSLPAQSACLKAIGCYGSGDTPGQNAKKLASVVAATT
MAGELSLLAALASNTLVQAHMAHNRKPATSK
>Attheya_septentrionalis
MTNTVASVPTVGERLDALIDTIDSLPSTHLYGAVVAVSVVFSFALLNWGGPSNHTREDAP
QKRRLSWEMRRSAASASAVSFKSGETEPQPKWHILRVLNYAAVGSFAASVVIFCRGASVY
LNDPATLLQFLVGWSLFLCYFFGFFGISFVDPDGMMMSYDPQQSQANNDPQDSTNCKSSS
AKKPLHPPAAATPVCSDGKASCKAPVVAAAALSSSAIKEMSDEQVAQLVVSNQVKDHMLE
KVLDPHRAVAVRRLAFDTKLASLDRGGALDELPSGPSLDYARVFGANCEIVVGYVPLPVG
MVGPLTLNNETVYVPMATTEGCLVASTNRGAKAICQGGGAHSCIVRDGITRAPCVRMKTA
MEAAKLKNWCEEPQHFAKLKAAFESTTSFGKLEQCNPTVAGRNVYLRLRCFSGDAMGMNM
VSKGSLAVIDLLKSEFPTLFLVALSGNMCTDKKPAAINWIEGRGKSVVVEAIIPKDVVRQ
TLKTTVQSICSTNIHKNLIGSAMAGAMGGFNAHAANIVTAIFLATGQDPAQNVESSNCIT
LMEETEEGNLWISCTMPSIEVGTVGGGTSLPAQSACLKAIGCKGGGAKPGQNAQQLAHVV
AAATMAGELSLLAALAANTLVAAHMQHNRKPTTTSK
>Thalassiosira_weissflogii
MAHPSSYERVSSPPPTIGKHIDAVIAKIDSLPSSYVYTLAIAASLTFSFYLLNSGGGSNS
MQMEPHGDVDRASAIKKPKQKSRRSLDEPQPKWHILKLTNYVATSGFLLSVLKFVSNASM
YLNDSTSLLQFLTIWSIFLCYFFGFFGISFIELDDFEQESHPQHQAPVSAINVSHAKHCN
AVKSGSLEPPVKVINVHPHSTSAPVCSDPASFRKPSSTIPGNLKDMSNEEIASLVLDDKI
KDHQLEKLLDPHRAVEVRRLKFDAQLQSLGRGGALAELPHKHDLDYKRVLGANCEIVVGY
VPIPVGMAGPITLNGESVYIPMATTEGCLVASTNRGCKAITQGSGAFSTIVRDGITRAPL
VRLPSAREAAEVLLWIEDENNFKILKEAFESTTSFGKLIEARPTVAGRNVYIRLRCFSGD
AMGMNMISKGSLAVIECLRRKFPQLSLAALSGNMCTDKKAAAINWIEGRGKSVVIEATIP
KEVVRSTLKTSVPAIVEANTNKNLIGSAMAATVGGFNAHAANNVTAVFLATGQDPAQNVE

SSNCITLMEETSDGDLWISCTMPSIEVGTVGGGTGLPAQSACLKAIGVKGGGDSPGDNSK
KLAHVVAAATMAGELSLMAALASNSLVAAHMAHNRKPVSK
>Odontella_Sinensis
MASSSVAYMTVGQRLDSFIESIDPSLSPTTKLYACVVCASVGFSFLLLNGRGSNVGGPDA
SGGPKSASKNESSSSGPSRRSGGGVSAASASSGGGRREPKWGVLKALNVVAALAFLISVI
RFASDASRHMSDSTSLLKFMSIWSALLCYFFGFFGISFVDAEGFSVAGEETKTTEKTTKK
AAGEEEDDDEDGGPGKKTAPAGSRSIPEKPQHANLHPPAPSAPVCSDPTSRAPITKKALP
PNLQGMPDNEIAALVLSGVVKDHQLEKLLSPARAVPVRRIVFERKLSSLNRGGALDELPH
EPSLDYGRVHGANCEIVVGYVPLPCGVIGPLTLDGETVYVPMATTEGCLVASANRGCKAI
SQGGGARSAVIRDGITRAPCVRMRSAMEAAELKLWCEDPANFARLKSAFESTTSFGKLLG
AHPTVAGRNVYLRLRCFSGDAMGMNMVSKGSLAVIELLRSTFPSLELVALSGNMCTDKKA
AATNWIEGRGKSVVVEATIPKDVVRATLKTTVPAMVNTNLQKNLVGSAMAGALGGFNAHA
ANIVTAIFLATGQDPAQNVESSNCITLLEETPTGDLWISCTMPSVEVGTVGGGTGLPAQA
SCLRAIGCKGGGANPGDNARKLARVVAASVLAGELSLLAALAANTLVQAHMIHNRKKPTA
GK
>Rhizosolenia_setigera
XNNPHFSATMTETIGQRXDALLESIDSVPSLQLYAAIVTSVVFSFVILNTGSHNAGLFQN
NEQQRTISSSQRKGSPTMKSNTANYTNTSFDRTKDNDQNQPQIKWYLLKTLNFAVAIGFV
ISITKFSSDASTYITDSISILKFLCAWSLCLCYFLGXFGMSFVDSEDLCSVPTAPPAGAT
SPVAAVHKKQVSKAASFSRPIHPPAMSTHPVCGTGEISSPTTTASLPDDIQSLSNEVAAS
LVVSNKIKDHTLEKHFGPFRAVXXRRLAFETKLASLGHSGALDDLPSGPSLDYKRVFGAN
CEIVVGYVPIPVGMCGPITLNGESVYIPMATTEGCLVASTNRGCKAISAGSGATSVILRD
GITRAPCLRMKSAKEAADLKLWCEEQENFLLLKXAFESTTSFGKLLSAEPTVSGKNVYXR
LRCFSGDAMGMNMISKGSLAVVEXLRELFPTLSLIALSGNMCTDKKAAATNWIEGRGKSV
VIEATIPKDVVTKILKTDVKSIVSTXLQKNLIGSAMAGALGGFNAHAANNVTAIFLATGQ
DPAQNVESSNCITLLEETEDGDLWICCTMPSIEVGTVGGGTGLPAQSSCLKMIGCKGGGE
TPGDNAKQLAHVVAAGTMAGELSLLAALASNTLVAAHMQHNRKPAQKK
>Coscinodiscus_wailesii
MTHAPANQHTTLGMRIDAVLQEIESISPTVLYSVILVASILFSMILLNTGGGRSTEPPRL
RDDVTKPSRKIQRFSDEPQPKWYILKFCNYAAVIGFAFSIFKFAIDATTYINDSTLLMKF
TVCWSLFMCYFFGFFGISFVDADEIEQRAVAGGFDGREGREVERKGILKGSITRKEHEYP
IHSPPQSIHPVCNPNNADIAKKQLPDKSPSSTRISDIKSLSDVEIASLVTSNKIKDHQLE
KLLDPRRAVDVRRIVVDSKLSSLGRGGALADLPSSPSLDYSRVFGANCEIVVGYVPIPVG
MVGPLTLNGETVYIPMATTEGCLVASTNRGCKAISAGSGAHSSIVRDGITRAPCLRLNST
REAADLMLWCEDESNFKLLKQAFESTTSFGKLLRVDPTIAGRNVYLRLKCFSGDAMGMNM
VSKGSLAVVDYLRTVFPTITLVALSGNVCTDKKAAAINWIEGRGKSVVVEATIPQHVVRS
TLKTTVEAIVSTNIQKNLIGSAMAGTVGGFNAHASNIVTAVFIATGQDPAQNVESSNCMT
LMEETPTGDLWISCTMPSIEVGTVGGGTGLPAQAACLKAMGCQGGAADKPGANAKQLAHV
VASATMAGELSLMAALAANTLVQAHMQHNRKPTIGKKA
>Skeletonema_marinoi
MTFSEPTTIGMRIDAVIASLESLPHTNPEILYASLIGASLMFSFLVLNSGGSSMALPDGG
DADSLNKPKKISNAKPVSSSDEPQPKWHILRLTNYVVTFGFLLSVLKFASNATTYLDDST
SLLQFLIIWSISMCYFFGFFGISFIELDELESEPQNHQHQMLRKPMPTEESAEPPVKVVK
MHPPASSAPVCTDPTSFKKPSPSIPSNIKELSNGEIASLVLQDKVKDHQLEKLLDPHRAV
EVRRLKVDNQLDSLGRGGALAELPHKHDLDYKRVLGANCEIVVGYIPIPVGMAGPITLNG
ESVYIPMATTEGCLVASTNRGCKAITQGSGAVSSILRDGITRAPCVRLPSAKEASEVFLW
IERPENFQKLKEVFESTTSFGKLLEARPSVAGKNVYIRLRCFAGDAMGMNMISKGSLAVI

ECLRQVFPNISLVALSGNMCTDKKAAAINWIEGRGKSVVIEATIPKGVVRSTLKTSVPAI
VEANVNKNLIGSAMAGVVGGFNAHASNNVTAVFLATGQDPAQNVESANCITLMEETPEGD
LWISCTMPSIEVGTVGGGTGLPAQAACLKAIGVKGGGENPGDNAKQLAHVVAAATMAGEL
SLMAALASNSLVAAHMTHNRKPASK
>Skeletonema_menzelii
MTSEATTIGMRIDAVIASLESLPHTNPEILYASVIGASLLFSFLVLNSGGSSMALPDGGD
ADSLNKPKISKAKLVSPSDEPQPKWHILRLTNYIVTFGFLLSVLKFASNATTYLDDSTSL
LQFVIIWSISMCYFFGFFGISFIELDELENEPQNHQHQILRKPMPTKESADPPVKVVKMH
PPASSAPVCIDPTSFKKPSPSIPSNIKELSNSEIASLVLQDKVKDHQLEKLLDPHRAVEV
RRLKVDVQLDSLGRGGALAELPHKHDLDYKRVLGANCEIVVGYVPIPVGMAGPITLNGES
IYIPMATTEGCLVASTNRGCKAISQGSGAVSSILRDGITRAPCVRLPSAKEASEVHLWIE
SPENFQKLKEVFESTTSFGKLLEARPSVAGKNVYIRLRCFAGDAMGMNMISKGSLAVIEC
LRQRFPNLSLVALSGNMCTDKKAAAINWIEGRGKSVVIEATIPKGVVRSTLKTSVPAIVE
ANLNKNLIGSAMAGVVGGFNAHASNNVTAVFLATGQDPAQNVESANCITLMEETPEGDLW
ISCTMPSIEVGTVGGGTGLPAQAACLKAIGVKGGGENPGDNAKQLAHVVAAATMAGELSL
MAALASNSLVAAHMTHNRKPASK
>Thalassiosira_miniscula
MPSSPTAPPPPPTLGMQIDAVIASLESISTPQLYGTVIAISLAFSFYLLNSGGGSSSPAL
QMMTNDTKEQQDADADSLRKPARAAATSSSSSSESSSEDRPEPRWHILRFTNYVAALGFL
VSVLQFASNASAYLNDSNSLLQFLTVWSLFVVYFVGFFGISFVELDDFVDGAQISSSVAR
QRAQQQQQQQQQQQSQNMTVAASQAKTPGRKSHKTDGSDSPPIKVVNVHPPATSAPVCSD
PKSFKKPSAASIPPNLKELPNSEIASLVLQDKIKDHQLEKLLDPHRAVQVRRLKFDARLS
ALGNGGALDELPHRHDLEYKRVLGANCEIVVGYVPIPVGMAGPVTLNGESVYIPMATTEG
CLVASTNRGCKAISQGSGASSTILKDGITRAPCVRLPSAKEAAEVALWIETPENFATLKE
AFESTTSFGKLLSARPTVAGKNVYVRLRCFSGDAMGMNMISKGSLAVIECLRQIFPALSL
VALSGNMCTDKKAAAMNWIEGRGKSVVIEATIPADVVRSTLKTSVTAIVEANLNKNLIGS
AMAGTVGGFNAHAANNVTAVFLATGQDPAQNVESSNCITLMEETPEGDLWMSCTMPSIEV
GTVGGGTGLPAQAACLRAIGAKGGGENPGDNARRLAHVVAAATMAGELSLMAALASNSLV
AAHMAHNRKPASK
>Thalassiosira_punctigera
XDTSELKGSKSSNRSPIXPSQNSESMGDATSAPPTVGMQIDAAIAQLENVSTTQLYAIVI
AASLAFSFYLLNSGGGSSALTIMNEKDGDADSLRKPEARKRKDRPRASTSSDEPEPRWHI
LRITNYVVAAGFLLSVLQFASNASTYLNDSTSLLQFLSVWSIFLCYFFGFFGISFIELDD
FVDQQPSQQQQQPSHKLKKAESISPDPPVKVVNVHPPSSSAPVCTDPTSFKKPPSNVPGN
LKDLPNSEIASFVLQDKIKDHQLEKLLDPHRAVEVRRLKFDAKLDSLGRGGALADLPHKH
DLDYKRVLGANCEIVVGYLPIPVGLAGPITLNGESVYIPMATTEGCLVASTNRGCKAISQ
GSGASSTILKDGITRAPCVRLPSAKEAAKVALWIGTPENFLTLKKAFESTTSFGKLLDAT
PTVAGRNVYIRIRCFSGDAMGMNMISKGSLAVIETLKKVFPDLSLLALSGNMCTDKKAAA
LNWIEGRGKSVVIEATIPKDVVRSTLKTSVQAIVEANVNKNLIGSAMAGTVGGFNAHAAN
NVTAVFIATGQDPAQNVESSNCITLMEETSEGDLWISCTMPSIEVGTVGGGTGLPAQSAC
LKVIGVKGGGENPGDNARQLAHVVAAATMAGELSLMAALASNSLVAAHMSHNRKPTSK
>Ditylum_brightwellii
KMATRLTETTDVTIGMRLDELISSIDTIPSTQLYMAVVVVSVIFSFLLLNSGGTSIEQEA
PPSSPPSTALKKITRQGPEPKWHILKILNYAAVSSFLVSVGCFASDASRHMNDSSSLFKF
LFGWSFFLCYFFGFFGISFVDADEIMEGQNFESSERSTSSKSKAVHPPAESTPVCTVPSS
FEKPSLSKTDLQSKSNEELSNMVLTGQIKDHQLEKLLDHHRAVDVRRLAFDSKLSSVGCG
GALTDLPSGPSLDYSRVFGANCEIVVGYVPLPVGMVGPLTLNGQSVYIPMATTEGCLVAS

TNRGCKAITQGSGATSVILRDGITRAPCVRMNSAKEAAELKLWCEMPANFATLKHAFEST
TGFGKLLSVEPTVAGKNAYLRLQCFAGDAMGMNMVSKGSLAVIDLLKSVFPTLVLVALSG
NMCTDKKAAATNWIHGRGKSVVVEAIIPQHVVRTTLKTTVQAIVQTNIHKNLIGSAMAGA
IGGFNAHAANNVTAVFLATGQDPAQNVESSNCITLLEETEDGDLWICCTMPSIEVGTVGG
GTSLPAQSACLKAIGCKGGGVNPGDNAKQLAHVVAAATMAGELSLLAALAANTLVQAHMT
HNRKPTTAKST
>Cylindrotheca_closterium
MASVISNYIDELSSTQLYTAIVGATVTLCVVLLGPAAGGNENLPNNMLGTTTTSNDNNKK
NAQQKQQPKWYLFKYINVGVFCMFVSSVAIFLWDASTYIHNGDRMTQFLVGWSLCLCYFF
GFFGVFFIHQDLLMNQNQNQQGDSSDIAENKQEMVPKTKEVKSVSPPPKAKVVHEPAACA
PVCSDPASFNSSSSSNKKAAISKDTDITGMTNDEIAQLVLDLQVKDHELEKRLDPFRAVT
VRRMIAQHKLQSVHSASSSAFLEDLPEGPSLDYSKVFGTNCEMVVGYVPLPVGMVGPLTL
NGESVYFPMATTEGCLVASTQRGAKAISQGTLGAQALIVKDGITRAPCVRMASAMEAAQL
KLWCQEAANLQVLKEAFESTTSFGKLLECHATVAGKNVYLRLVCFSGDAMGMNMVSKGSL
KVIETLQNIFPNLELVALSGNMCTDKKAAATNWLHGRGKSVVVEAVIPKDVVKKTLKTTV
PALVSTNQNKNLIGSAMAGAMGGFNAHASNIVTAIFLATGQDPAQNVESANCITLMEQVE
ETGDLWISCTMPSIEVGTVGGGTTLPAQAACLEILGCKGGSRENPGKNAQKLALVVAAAT
MAGELSLMSALAANTLVQAHMKHNRKKTV
>Nitzschia_punctata
MAATFDTMPTTIGMKLDAMIAEIDNLSSTQLYGVIVAATVVLCVVLLGTGHSNLDLQHSN
NNDPLLKKQPAVAPSGNIKQPRWHIFKWINYLAVAAFLWSVCTFCLNASQYLHHEESQGV
LVKFLLGWSVFLLYFFGFFGVSLIHEDIPKEEAGAASSAANRLSSVSQSNKTKTVETSSK
NKALHGAAACTPVCSDPSSFKVSSSVPSNIKELADEDVADLVLKNKVKDHELEKRLDPFR
AVTVRRMTANRKLASVLPQNKPNVLDKLPATPSLDYSKVHGANCEIVVGYVPLPVGLVGP
LSLNNETVYYVPMATTEGCLVASTNRGAKAITQGGGAQARIVRDGITRAPCVRMESAMGAA
DLKVWCEKPENFARLKQAFEGTTSFGKLQACHPTVAGKNVYLRLVCFSGDAMGMNMVSKG
SLAVIETLQKEFPSLQLVALSGNMCTDKKAAATNWLQGRGKSVVVEATIPKDVVRTTLKT
TVAALVHTNMHKNLIGSAMAGSLGGFNAHASNIVTAVFLATGQDPAQNVESSNCITLMEE
TDEGDLWISCTMPSIEVGTVGGGTSLEAQAACLEAIGCKGGGATPGENAKKLATVVAAAT
MAGELSLLAALAANTLVQAHMTHNRKSNKK
>Nitzschia_sp.
VLSQVDELSSTQLYGLIVAATVTLCVFLLGTGSNTDVEFAATNSKMKTGTNDDGLKKPNA
STNNTASTTRQPRWHIFKWVNYLASAAFLSSVGMFCMNASQYLHHESKGVLVQFLVGWSV
FLMYFFGFFGISLIHEDIPKDNQDDQGIEQPRNTTTRYAAGSSSTTAAAKKQALHTAAPS
APVCSDPASFKASSSSSVPSNLKELPDEDIANLVLMNKLKDHELEKRLDPFRAVTVRRLV
VNQKLSTLLTDGKTNQPVTNVLEKLPSTPSLDYSRVFGANCEIVVGYVPLPVGLVGPLSL
NDETVYVPMATTEGCLVASTNRGAKAITQGGGAQARIVRDGITRAPCVRMASAMEAADLK
VWCEEPQNFAVLKQAFESTTSFGKLQACNPTVAGKNVYLRLVCFSGDAMGMNMVSKGSLA
VIETLQAKFPSLQLVALSGNMCTDKKAAATNWLHGRGKSVVVEAIIPKDVXPWNSQNNSL
SLNDETVYVPMATTEGCLVASTNRGAKAITQGGGAQARIVRDGITRAPCVRMASAMEAAD
LKVWCEEPQNFALLKQAFESTTSFGKLQACNPTVAGKNVYLRLVCFSGDAMGMNMVSKGS
LAVIETLQAKFPSLQLVALSGNMCTDKKAAATNWLHGRGKSVVVEAIIPKDVVRGTLKTT
VDALIFTNTNKNLIGSAMAGSIGGFNAHASNIVTAVFLATGQDPAQNVESSNCITLMEPT
DDGDLWISCTMPSIEVGTVGGGTSLPAQSACLEAIGCKGGGATAGENAQKLARVVAAATM
AGELSLLAALAANTLVQAHMQHNRKPAPKKELTLTPSFVQNNTPIVPFLRKAHLERTYRS
VLGCSQIVIRKSRAPCVRMASAMEAADLKVWCEEPQNFAVLKQAFESTTSFGKLQACNPT
VAGKNVYLRLVCFSGDAMGMNMVSKGSLAVIETLQAKFPSLQLVALSGNMCTDKKAAATN

WLHGRGKSVVVEAIIPKDVVRGTLKTTVDALVHTNTNKNLIGSAMAGSIGGFNAHASNIV
TAVFLATGQDPAQNVESSNCITLMEPTDDGDLWISCTMPSIEVGTVGGGTSLPAQSACLE
AIGCKGGGATPGENAQKLATVVAAATMAGELSLLAALAANTLVQAHMQHNRKPAPKK
>Pseudonitzschia_pungens
MAQTMGTMATIGTRIDNVIAQFDQLPSTQLYGLIVAATVGLCVVLLGTGNSNFELHHQRQ
KQLQQNNDDLKKPKMAAMPSGRQPRWHIFKWINFVAVGAFLCSVFIFCSNASRYLHHESQ
GVLVQFLVGWSVFLMYFFGFFGVSLIHDDIPREEEESLPSKPQTKSVPTNTSAPKKTVHP
PAACTPVCSDPSSFKSSKPSVPENLKELSDDEIAALVLGNKVKDHMLEKLLDPFRAVTVR
RIACNQKLASVLGREDKSNVLEKLPSEPSLDYSRVFGANCEIVVGYVPLPVGLVGPLTIN
DESVYVPMATTEGCLVASSNRGAKAITQGGGARAKIVRDGITRAPCLRMNTAMEAADLKI
WCEKPQNFAILKQAFESTTSFGKLKECNPTVAGKNVYLRLVCFSGDAMGMNMVSKGSLAV
IETLQKYFPTCQLVALSGNMCTDKKAAATNWLYGRGKSVVVECVIPKEVVRTTLKTTVSA
LVHTNLNKNLIGSAMAGAIGGFNAHASNIVTAVFLATGQDPAQNVESSNCITLMEEEVNG
DLWMCCTMPSIEVGTVGGGTSLPAQAACLEAIGCKGGGVTPGANAKKLATVVAAATMAGE
LSLLAALAANTLVQAHMAHNRKPASKK
>Saccharomyces_cerevisiae
MPPLFKGLKQMAKPIAYVSRFSAKRPIHIILFSLIISAFAYLSVIQYYFNGWQLDSNSVF
ETAPNKDSNTLFQECSHYYRDSSLDGWVSITAHEASELPAPHHYYLLNLNFNSPNETDSI
PELANTVFEKDNTKYILQEDLSVSKEISSTDGTKWRLRSDRKSLFDVKTLAYSLYDVFSE
NVTQADPFDVLIMVTAYLMMFYTIFGLFNDMRKTGSNFWLSASTVVNSASSLFLALYVTQ
CILGKEVSALTLFEGLPFIVVVVGFKHKIKIAQYALEKFERVGLSKRITTDEIVFESVSE
EGGRLIQDHLLCIFAFIGCSMYAHQLKTLTNFCILSAFILIFELILTPTFYSAILALRLE
MNVIHRSTIIKQTLEEDGVVPSTARIISKAEKKSVSSFLNLSVVVIIMKLSVILLFVFIN
FYNFGANWVNDAFNSLYFDKERVSLPDFITSNASENFKEQAIVSVTPLLYYKPIKSYQRI
EDMVLLLLRNVSVAIRDRFVSKLVLSALVCSAVINVYLLNAARIHTSYTADQLVKTEVTK
KSFTAPVQKASTPVLTNKTVISGSKVKSLSSAQSSSSGPSSSSEEDDSRDIESLDKKIRP
LEELEALLSSGNTKQLKNKEVAALVIHGKLPLYALEKKLGDTTRAVAVRRKALSILAEAP
VLASDRLPYKNYDYDRVFGACCENVIGYMPLPVGVIGPLVIDGTSYHIPMATTEGCLVAS
AMRGCKAINAGGGATTVLTKDGMTRGPVVRFPTLKRSGACKIWLDSEEGQNAIKKAFNST
SRFARLQHIQTCLAGDLLFMRFRTTTGDAMGMNMISKGVEYSLKQMVEEYGWEDMEVVSV
SGNYCTDKKPAAINWIEGRGKSVVAEATIPGDVVRKVLKSDVSALVELNIAKNLVGSAMA
GSVGGFNAHAANLVTAVFLALGQDPAQNVESSNCITLMKEVDGDLRISVSMPSIEVGTIG
GGTVLEPQGAMLDLLGVRGPHATAPGTNARQLARIVACAVLAGELSLCAALAAGHLVQSH
MTHNRKPAEPTKPNNLDATDINRLKDGSVTCIKS
>Arabidopsis_thaliana
MDLRRRPPKPPVTNNNNSNGSFRSYQPRTSDDDHRRRATTIAPPPKASDALPLPLYLTNA
VFFTLFFSVAYYLLHRWRDKIRYNTPLHVVTITELGAIIALIASFIYLLGFFGIDFVQSF
ISRASGDAWDLADTIDDDDHRLVTCSPPTPIVSVAKLPNPEPIVTESLPEEDEEIVKSVI
DGVIPSYSLESRLGDCKRAASIRREALQRVTGRSIEGLPLDGFDYESILGQCCEMPVGYI
QIPVGIAGPLLLDGYEYSVPMATTEGCLVASTNRGCKAMFISGGATSTVLKDGMTRAPVV
RFASARRASELKFFLENPENFDTLAVVFNRSSRFARLQSVKCTIAGKNAYVRFCCSTGDA
MGMNMVSKGVQNVLEYLTDDFPDMDVIGISGNFCSDKKPAAVNWIEGRGKSVVCEAVIRG
EIVNKVLKTSVAALVELNMLKNLAGSAVAGSLGGFNAHASNIVSAVFIATGQDPAQNVES
SQCITMMEAINDGKDIHISVTMPSIEVGTVGGGTQLASQSACLNLLGVKGASTESPGMNA
RRLATIVAGAVLAGELSLMSAIAAGQLVRSHMKYNRSSRDISGATTTTTTTT
>Homo_sapiens
MLSRLFRMHGLFVASHPWEVIVGTVTLTICMMSMNMFTGNNKICGWNYECPKFEEDVLSS

DIIILTITRCIAILYIYFQFQNLRQLGSKYILGIAGLFTIFSSFVFSTVVIHFLDKELTG
LNEALPFFLLLIDLSRASTLAKFALSSNSQDEVRENIARGMAILGPTFTLDALVECLVIG
VGTMSGVRQLEIMCCFGCMSVLANYFVFMTFFPACVSLVLELSRESREGRPIWQLSHFAR
VLEEEENKPNPVTQRVKMIMSLGLVLVHAHSRWIADPSPQNSTADTSKVSLGLDENVSKR
IEPSVSLWQFYLSKMISMDIEQVITLSLALLLAVKYIFFEQTETESTLSLKNPITSPVVT
QKKVPDNCCRREPMLVRNNQKCDSVEEETGINRERKVEVIKPLVAETDTPNRATFVVGNS
SLLDTSSVLVTQEPEIELPREPRPNEECLQILGNAEKGAKFLSDAEIIQLVNAKHIPAYK
LETLMETHERGVSIRRQLLSKKLSEPSSLQYLPYRDYNYSLVMGACCENVIGYMPIPVGV
AGPLCLDEKEFQVPMATTEGCLVASTNRGCRAIGLGGGASSRVLADGMTRGPVVRLPRAC
DSAEVKAWLETSEGFAVIKEAFDSTSRFARLQKLHTSIAGRNLYIRFQSRSGDAMGMNMI
SKGTEKALSKLHEYFPEMQILAVSGNYCTDKKPAAINWIEGRGKSVVCEAVIPAKVVREV
LKTTTEAMIEVNINKNLVGSAMAGSIGGYNAHAANIVTAIYIACGQDAAQNVGSSNCITL
MEASGPTNEDLYISCTMPSIEIGTVGGGTNLLPQQACLQMLGVQGACKDNPGENARQLAR
IVCGTVMAGELSLMAALAAGHLVKSHMIHNRSKINLQDLQGACTKKTA
>Oryza_sativa
MDVRRGGGGGRIVGAARRALTWGALPLPMRITNGLAMVSLVLSSCDLLRLCSDRERPLGG
REFATVVYLVSLFAHPDAPATTTGDDDDGQGGSRRARPAAAEPAPMHGHGGGMMEADDEE
IVAAVASGALPSHRLESRLGDCRRAARLRREALRRVTGRGVEGLPFDGMDYQAILGQCCE
MPVGYVQLPVGVAGPLLLDGREYHVPMATTEGCLVASVNRGCRAISASGGAFSVLLRDAM
SRAPAVKLPSAMRAAELKAFAEAPANFELLAAVFNRSSRFGRLQDIRCALAGRNLYMRFS
CITGDAMGMNMVSKGVENVLGYLQNVFPDMDVISVSGNYCSDKKPTAVNWIEGRGKSVVC
EAIIKGDVVQKVLKTTVEKLVELNIIKNLAGSAVAGALGGFNAHASNIVTALFIATGQDP
AQNVESSQCITMLEEVNDGDDLHISVTMPSIEVGTIGGGTCLASQAACLNLLGVKGSNHG
SPGANAKRLATIVAGSVLAGELSLLAALASGHLVKSHMMYNRSSKDVAKAAS
>Solanum_lycopersicum
MDVRRRSEEPVYPSKVFAADEKPLKPHKKQQQQQEDKNTLLIDASDALPLPLYLTTNGLF
FTMFFSVMYFLLSRWREKIRNSTPLHVVTLSELGAIVSLIASVIYLLGFFGIGFVQTFVS
RGNNDSWDENDEEFLLKEDSRCGPATTLGCAVPAPPARQIAPMAPPQPSMSMVEKPAPLI
TSASSGEDEEIIKSVVQGKIPSYSLESKLGDCKRAASIRKEVMQRITGKSLEGLPLEGFN
YESILGQCCEMPIGYVQIPVGIAGPLLLNGKEFSVPMATTEGCLVASTNRGCKAIYASGG
ATCILLRDGMTRAPCVRFGTAKRAAELKFFVEDPIKFESLANVFNQSSRFARLQRIQCAI
AGKNLYMRLCCSTGDAMGMNMVSKGVQNVLDYLQNEYPDMDVIGISGNFCSDKKPAAVNW
IEGRGKSVVCEAIITEEVVKKVLKTEVAALVELNMLKNLTGSAMAGALGGFNAHASNIVS
AVFIATGQDPAQNIESSHCITMMEAVNDGKDLHISVTMPSIEVGTVGGGTQLASQSACLN
LLGVKGANREAPGSNARLLATVVAGSVLAGELSLMSAISSGQLVNSHMKYNRSTKDVTKA
SS
>Thalassiosira_rotula
MVVATSAPPTVGMQIDAAIASLETLSTTQLXATVIAVSLXFSFYLLNSGGESSALTMMNE
SSSDSLRKPKXDRKRMKTQQQQSREETDEPEPRWHILRITNYVVATGFTLSVLQFASNAS
TYLNDSTSLLQFLMVWSLFLCYFFGFFGISFIELDDFVDQQQPSQQQPSQQTQVAPSRQL
KKAESSDPPVKVVNIHPPASSAPVCTDPTSFKKPSSKIPSNLKELPNSEIASLVLQDKIK
DHQLEKLLDPHRAVEVRRLKFDAKLDSLGCGGALTELPHKHALDYKRVLGANCEIVVGYI
PIPVGMAGPITLNGESVYIPMATTEGCLVASTNRGCKAISQGSGASSTILKDGITRAPCV
RLPSAREAAEVALWIGKTXNFLSLKEAFESTTSFGKLLDATPTVAGKNVYIRLRCFSGDA
MGMNMISKGSLAVIDALRQIFPRLSLLALSGNMCTDKKAAAMNWIEGRGKSVVIEATIPQ
DVVRSTLKTSVRAITEANVNKNLIGSAMAGTVGGFNAHAANNVTAVFLATGQDPAQNVES
SNCITLMEETPEGDLWISCTMPSIEVGTVGGGTGLPAQSACLKAIGVKGGGENPGDNAKQ

LAHVVAAATMAGELSLMAALASNSLVAAHMAHNRKPASK
>Pseudonitzschia_delicatissima
CKRTFPSXRSNTDLHPSNKNMAETMTPMPTIGMQLDAMIAKVDALPSTQLYGLIVAVTVG
LCVVVLGTGNSNLEMEQQQQDLKKPKMVVVDGGKQPNWHLFKMINYIAVAAFLYSVFIFC
SNASQYLHHGSQGVLAQFLVGWSVFLMYFFGFFGVSLIHDDIPSDTDSVPSQPQTKSVAP
KPVHKPAACTPVCSDPSSFKSSKSSVPENIKELGDEEIADLVLSNKVKDHMLEKLLDPFR
AVKVRRIACNQKLNSVLATNSTNVLDKLPSEHDLDYGRVYGANCEIVVGYVPLPVGLVGP
LTINDESFYVPMATTEGCLVASSNRGAKAIVQGGGARARIVRDGITRAPCLRMNTAMEAA
DLKIWCEEPQNFAILKQAFESTTSFGKLQSCNPTVAGKNVYLRLVCFSGDAMGMNMVSKG
SLAVIEKLQEHFPTCQLVALSGNMCTXKKAAATNWLHGRGKSVIVECIIPKEVVRTTLKT
TVRALVHTNINKNLIGSAMAGAIGGFNAHASNIVTAIFLATGQDPAQNVESSNCITLMEE
QDNGDLWMCCTMPSIEVGTVGGGTSLPAQAACLEAIGVKGGGVVPGANAKKLATVVAAAT
MAGELSLLAALAANTLVQAHMAHNRKPAAKK
>Pseudonitzschia_arenysensis
MAETMTPMPTIGMQLDAMIAQIDALPSTQLYGLIVAATVGLCVVVLGTGNSNLEMEQHDL
KKPKMVPVDGGKQPNWQIFKVINYIAVAAFLYSVFMFCSNASKYLHHESQGVLAQFLVGW
SVFLMYFFGFFGVSLIHEDIPSEVDSVPSQPKSVAPTKPVHPPAACAPVCSDPSSFKAKK
SCVPENIKELGDEEIADLVLANKVKDHMLEKLLDPFRAVTVRRIACNRKLNSVHGNTSNV
LDKLPSEHALDYSRVYGANCEIVVGYVPLPVGLVGPLTINDESFYVPMATTEGCLVASSN
RGAKAICQGGGAKARIVRDGITRAPCLRMNSAMEAADLKIWCEKPANFAILKKAFESTTS
FGKLIECNPTVAGKNVYLRLVCFSGDAMGMNMVSKGSLAVIEKLQEYFPSCQLVALSGNM
CTDKKAAATNWLHGRGKSVVVECIIPKEVVRTTLKTTVAALVHTNVNKNLIGSAMAGAIG
GFNAHASNIVTAIFLATGQDPAQNVESSNCITLMEKQDNGDLWMCCTMPSIEVGTVGGGT
SLPAQAACLEAIGCKGGGATPGANAKQLATVVAAATMAGELSLLAALAANTLVQAHMAHN
RKPAAKK
>Fragilariopsis_kerguelensis
MASTEVMPTLGMRLDAMISHIDRLPSSQLYGVIVAATVGLCVVLLGTGNSNLELQQQQRQ
RQQQMMNVDDLKKPINNRIANVGGKQPKWHIFKWINYLAVGAFLWSVYTFCSNASQYLHH
ESQGVLVQFLVGWSVFLLYFFGFFGISLIHEDIPKEEDETTTMTVSKNIVSRKPYNASTN
STTTMMKKKNSIHPPASCAPVCSDPTSFKSSGASLSSNSVNLEELADEEIANLVLNNKVK
DHELEKRLDPFRAVTVRRIAFNQKIASVLSDNTNKQNNINNTANVLDKLPSTPSLDYSRV
YGANCEIVVGYVPLPVGLVGPLTINDETVYIPMATTEGCLVASSNRGAKAITQGSGAKAR
IVRDGITRAPCIRMRSAMEAADLKLWCEEPSNFLILKQAFESTTNFGKLKECNPTVAGKN
VYLRLVCFSGDAMGMNMVSKGSLAVIETLQNEFPSLQLVALSGNMCTDKKAAATNWLNGR
GKSVVVEAVIPREVVEKTLKTTVKALVHTNINKNLIGSAMAGAIGGFNAHASNIVTAIFL
ATGQDPAQNVESSNCITLIEETDDNDLWISCTMPSIEVGTVGGGTSLEAQSACLEAIGCK
GGGATPGENARKLATVVAAATMAGELSLLAALAANTLVQAHMVHNRKPASNK
>Leptocylindrus_danicus
MAQTADPMTIGTRLDALIASGAEYLNNASQAQVCATLFVSSVAFSFALLNCGKGAHGSSL
DPTLKPLYKNTMEPIKTKVKSPDGSREPRWYIFKMLNYTAVATFSTSVLHFILYSDVYMN
NDAQMMKLMGAWTLFVLYFFGFFGVSFVDTDDHLEGSSPAEDEISEMTADQASPSAVVAV
AKQMPVKAAAPIHPPAPSHPVCSDKNLTKDALTSSSATPAVKKSSSLSLDLQSMTNEELA
DLVLTNKMKDHQLETKLNPTRAVPIRRLVFEKKLASLGHAKSLDELPYEHSLSYERVFGA
NCEIVVGYVPLPVGMVGPCTLNGESVYIPMATTEGCLVASTNRGCKAITAGGGAVSTLLR
DGITRAPCLRFESAAEAAALALWAQEPHNFAKLKAAFESTTSFGKLLSATPTVAGKNCYL
RLKCFSGDAMGMNMVSKGSLAVVDLLRQHFPTLKLVALSGNMCTDKKPAAINWIEGRGKS
VVVEATIPKDIVRTVLKTTVKAIVDTNLQKNLIGSAMSGSVGGYNAHASNIVTAVFLATG

QDPAQNVESSNCITIMEETDDGDLWISCTMPSIEVGTVGGGTSLPAQAACLKAIGVKGGG
DIPGGNARKLAHVVAVATMAGELSLLAALAANTLVQAHMQHNRKPATPAKK
>Chaetoceros neogracile
METQIPTIGMRIDSLISYFDAIPNTSKAFVIICCLFSFFLLNSSPNPAATLNTSTYPGCD
VTKAKTSNKGSSACNTVSKSSNCESEPQPKWHILKLLNVIAVIGLLTSFFWFASNASTYI
NDSNALLKFMALWSGFLCYFFGFFGISFIDVEELERGSSEEEEKQRTNECQKSLFVHKQL
PVHPPASCTPVCSDNLKISTDKPSKPTTFTPSSNSTNTNQKTDIKSLSNSEIVSMVLSNK
IKDHQLEKLLDPYRAVIVRRATFDQKLSTLNQGDALNDLPYEHDLEYSRVFGANCETVIG
YVPLPVGMVGPLTLNGQTVYFPMATTEGCLVASTNRGCKAISQGSGASSTITRDGITRAP
CIRMKSAKEAAELKIWCEIPQNFLTLKAAFESTTSFGKLQSAQVTVAGKNAYVRLCCFSG
DAMGMNMVSKGSLAVIDLLKEKFPTLDLLALSGNMCTDKKAAAINWIEGRGKSVVVESII
PKDVVHATLKTDVKKIVTVNINKNLIGSAMAGSIGGFNAHASNIVSAVFLATGQDPAQNV
ESSNCITLMEETEEGDLWVSCTMPSIEVGTVGGGTGLPAQSAGLKIIGCKGGGEQPGANA
KKLAHVVASAVMAGGPCFFF
>Chaetoceros dichaeta
QHQIQNAPNSGMRSSVVNPPPSLHAPARHTPVCTDPPPNPKPSPTTPLPPPESTSDQAWA
DMVLTNQVKDHQLEKILNPHRAVVVRRLIFQSKLGSLATDESCTSPLDELPYQHSLDYTR
VHGANCETVVGYIPLPVGMVGPLTLNNQTVYFPMATTEGCLVASTNRGCKAISQGSGAIS
TIVRDGITRAPCLKMRSAREACELKLWCELPSNFCKLKEAFESTTGFGKLQSATTMIAGR
NAYVRLCCFSGDAMGMNMVSKGTLAVVDLLRSIFPTLELVALSGNVCTDKKPSAINWIEG
RGKSVVVEATIPKDVVHTTLKTEVKTIVRVNIDKNLIGSALAGSIGGFNAHAANIVTALF
LATGQDPAQNVESSNCMTLMEETDNGDLWISCTMPSIEVGTVGGGTGLPAQKACLKVIGC
QGGGVKPGENSRQLAHVVASATMAGELSLMAALSANTLVQAHMQHNRKPATTSAVDSKQ*
>Chaetoceros affinis
MPMEDIQSLSNDEIASLVLTNKIKDHRLEKLTDPDRAVIIRRAVYSHKLATLPTSNSTTS
NSPIDALPHLHSLNYSKVYGANCETVIGYVPLPVGIVGPITLNNQSVYFPMATTEGCLVA
STNRGCKAITEGGGAVSVITRDGITRAPCVRMESAKDAALLKLWCEDTTNQSENFVQLKK
AFESTTSFGKLEAVNVSIAGRNAYIRLRCFSGDAMGMNMVSKGSLAVIDYLKTIFPSLVL
VALSGNVCTDKKAAAMNWIEGRGKSVVVEATIPRDVVVKTLKTTVKAMVSVNINKNLIGS
ALAGVVGGFNAHASNIVSAVFLATGQDPAQNVESSNCMTLMEETDSGDLWISCTMPSIEV
GTVGGGTGLAAQAACLGIVGCRGGGENPGDNAKQLAHVVAAAVMAGGVSLLGA
